# L‐arginine promotes gut hormone release and reduces food intake in rodents

**DOI:** 10.1111/dom.12644

**Published:** 2016-04-01

**Authors:** A. Alamshah, A. K. McGavigan, E. Spreckley, J. S. Kinsey‐Jones, A. Amin, I. R. Tough, H. C. O'Hara, A. Moolla, K. Banks, R. France, G. Hyberg, M. Norton, W. Cheong, A. Lehmann, S. R. Bloom, H. M. Cox, K. G. Murphy

**Affiliations:** ^1^ Section of Endocrinology and Investigative Medicine, Department of MedicineImperial College LondonLondonUK; ^2^ Wolfson Centre for Age‐Related Diseases, Institute of Psychiatry, Psychology and NeuroscienceKing's College LondonLondonUK; ^3^ AstraZeneca R&DMölndalSweden; ^4^ Institute of Neuroscience and PhysiologyThe Sahlgrenska Academy at the University of GothenburgGothenburgSweden

**Keywords:** animal pharmacology, body composition, energy regulation, GLP‐1, obesity therapy

## Abstract

**Aims:**

To investigate the anorectic effect of L‐arginine (L‐Arg) in rodents.

**Methods:**

We investigated the effects of L‐Arg on food intake, and the role of the anorectic gut hormones glucagon‐like peptide‐1 (GLP‐1) and peptide YY (PYY), the G‐protein‐coupled receptor family C group 6 member A (GPRC6A) and the vagus nerve in mediating these effects in rodents.

**Results:**

Oral gavage of L‐Arg reduced food intake in rodents, and chronically reduced cumulative food intake in diet‐induced obese mice. Lack of the GPRC6A in mice and subdiaphragmatic vagal deafferentation in rats did not influence these anorectic effects. L‐Arg stimulated GLP‐1 and PYY release in vitro and in vivo. Pharmacological blockade of GLP‐1 and PYY receptors did not influence the anorectic effect of L‐Arg. L‐Arg‐mediated PYY release modulated net ion transport across the gut mucosa. Intracerebroventricular (i.c.v.) and intraperitoneal (i.p.) administration of L‐Arg suppressed food intake in rats.

**Conclusions:**

L‐Arg reduced food intake and stimulated gut hormone release in rodents. The anorectic effect of L‐Arg is unlikely to be mediated by GLP‐1 and PYY, does not require GPRC6A signalling and is not mediated via the vagus. I.c.v. and i.p. administration of L‐Arg suppressed food intake in rats, suggesting that L‐Arg may act on the brain to influence food intake. Further work is required to determine the mechanisms by which L‐Arg suppresses food intake and its utility in the treatment of obesity.

## Introduction

High protein diets promote satiety and weight loss 1, 2, but the exact mechanisms mediating these effects are unclear. Evidence suggests, however, that protein is sensed within the gastrointestinal tract, modulating appetite‐regulating pathways 3.

Mechanisms proposed to mediate the effects of high protein diets on food intake, include increased thermogenesis, intestinal gluconeogenesis and changes in gut hormone profiles 4. Such mechanisms may be instigated by the sensing of the amino acids produced by protein digestion. Rodents adapt their diet to balance amino acid intake 5. Different types of protein can result in different levels of satiety 6, perhaps reflecting their different amino acid compositions. The recent discovery of promiscuous L‐amino acid‐sensing G‐protein‐coupled receptors and their expression in the gastrointestinal tract has driven speculation that these receptors are involved in amino acid sensing and the regulation of food intake. These receptors include the calcium‐sensing receptor (CaSR), the T1R1‐T1R3 umami taste receptor complex and the G‐protein‐coupled receptor family C group 6 member A (GPRC6A) 7.

Amino acids may be sensed in the gut to promote the release of anorectic gut hormones 8. The aromatic amino acids L‐phenylalanine and L‐tryptophan induce the release of cholecystokinin (CCK) from isolated I‐cells 9. The anorectic gut hormones glucagon‐like peptide‐1 (GLP‐1) and peptide YY (PYY) (3‐36) are released from enteroendocrine L‐cells in response to nutrients, including amino acids 10. Ingestion of a high protein meal alters circulating GLP‐1 and PYY levels and promotes their release in both humans and rodents 11, 12. These hormones may act directly on appetite‐regulating areas of the brain, but may also have indirect effects. The vagus nerve is one of the major extrinsic nerves, with a key role in the gut–brain axis. Evidence suggests that vagal signalling is involved in the regulation of food intake, and may play a role in gut hormone‐mediated satiety. Vagal afferents relay mechanosensory and chemosensory signals from the gut to the nucleus of the solitary tract within the brainstem. In addition, specific gut hormones, in particular CCK, but also ghrelin, PYY(3‐36) and GLP‐1, have been reported to exert their effects on appetite and food intake via vagal afferents 13.

The ability of specific L‐amino acids, including L‐arginine (L‐Arg), to stimulate GLP‐1 and PYY release has been studied previously *in vitro* 14, 15. L‐Arg, a conditionally essential amino acid, is derived from the diet, endogenous synthesis and protein turnover 16. L‐Arg has a well‐characterized effect as a secretagogue promoting insulin release from pancreatic β‐cells 17. Oral L‐Arg can also stimulate insulin secretion by promoting GLP‐1 release, improving glucose tolerance in mice 18. L‐Arg is a potent agonist of GPRC6A 19, and it has been suggested that GPRC6A activity is necessary for some effects of L‐Arg on glucose homeostasis 20. Furthermore, GPRC6A was required for ornithine‐induced GLP‐1 release from an *in vitro* model 21, suggesting that GPRC6A may play a role in L‐Arg‐mediated hormone release. In addition, L‐Arg can stimulate growth hormone release from the pituitary, although the mechanism is unclear 22.

L‐Arg thus has established effects on hormone release and metabolism. Recent evidence suggests that L‐Arg may also be involved in the regulation of food intake 23; therefore, we investigated the effect of L‐Arg on gut hormone release and energy homeostasis in rodents, and explored the potential mechanisms mediating its effects on gut function and food intake.

## Materials and Methods

### Animals

Male C57BL/6 mice, 8–10 weeks (Harlan, Bicester, UK) and male Wistar rats (200–250 g) (Charles River, Margate, UK) were individually housed under controlled temperature (21–23 °C) and humidity on a 12 h light : 12 h darkness cycle. All the animals had *ad libitum* access to standard chow RM1 (SDS, Witham, UK) and water, and were randomized by body weight, unless stated otherwise. The GPRC6A knock‐out (GPRC6a‐KO) model used in the present studies was generated by the trans‐NIH Knock‐Out Mouse Project (KOMP) and obtained from the KOMP Repository. The deleted region completely covers the GPRC6a locus 24, and thus this model differs from others previously described 25, 26. The glucose homeostasis phenotype of the knock‐out model was assessed before performing feeding studies to address the conflicting reported phenotypes of other GPRC6a KO models 25, 27. All animal procedures were approved and performed under the UK Home Office Animals (Scientific Procedures) Act 1986.

### Feeding Studies

Animals were randomized by body weight and acclimatized to the procedures before all studies. Because of the basic nature of L‐Arg solution, L‐Arg monohydrochloride (L‐Arg·HCl) neutral salt was used in all experiments. For the fasted studies, animals were fasted for 16 h overnight before receiving water or L‐Arg·HCl (Sigma, Poole, UK), at doses stated (Table S1, File S1), in the early light phase by either oral gavage (o.g.) or intraperitoneal (i.p.) injection. For *ad libitum*‐fed animal studies, the same administration procedure was used without fasting in either the early light phase or at the onset dark phase. For the study investigating the role of gut hormones in mediating the effect of L‐Arg on food intake, fasted or *ad libitum*‐fed mice were given simultaneous i.p. administration of 400 nmol/kg exendin 9‐39 (GLP‐1R antagonist) and BIIE0246 (Y_2_ receptor antagonist) at 5.26 µmol/kg 15 min before the o.g. administration of water or 24 mmol/kg L‐Arg. Animals were returned to their cages, with pre‐weighed amounts of standard chow diet and food intake measured 1, 2, 4, 8 and 24 h after administration. The GPRC6a‐KO mice feeding studies used a crossover design, in which GPRC6a‐KO and wild‐type (wt) mice received both control and L‐Arg treatments in random order on separate occasions separated by at least 3 days; accordingly, food intake was compared and analysed using a paired analysis approach. A summary of all feeding studies, including doses, species and time of day of the study, is provided in Table S1, File S1.

### Energy Expenditure Studies

Mice were individually housed in a 24‐chamber open‐circuit comprehensive laboratory animal monitoring system (CLAMS; Columbus Instruments, OH, USA) and acclimatized for 24 h to generate stable reference data. They were then fasted for 16 h overnight and subsequently received water or 24 mmol/kg L‐Arg o.g. (n = 12/group) at 09:00 hours (early light phase). The mice continued to be fasted for the subsequent 8 h, to examine the effects of L‐Arg on energy expenditure, independent of effects on food intake, before food was returned at 17:00 hours. Metabolic variables (VO_2_ and VCO_2_) and the respiratory exchange ratio (RER) were measured every 24 min for 24 h after treatment administration, and values normalized to body weight 28.

### Chronic Feeding Studies in Mice

Male mice, aged 6–8 weeks, were group‐housed (five per cage) with *ad libitum* access to water and a 60% high fat diet (Research Diets, New Brunswick, NJ, USA) for 8 weeks. The mice were then individually housed and given 1 week to acclimatize before the study started, remaining on the high fat diet. Mice were given water or 16 mmol/kg L‐Arg o.g. (n = 9 per group) twice daily throughout the dark phase at 19:00 hours and then 01:00 hours for five nights. Body weight and food intake were measured daily at the beginning of the dark phase and at 1 h after the first daily gavage.

### Subdiaphragmatic Vagal Deafferentation Surgery in Rats

Subdiaphragmatic vagal deafferentation (SDA) was carried out in rats, as previously described 29, 30, as it results in more accurate deafferentation and lower morbidity than in mice. The effect of oral administration of water or 16 mmol/kg L‐Arg (n = 9–10, crossover) on food intake was then studied in overnight fasted rats during the early light phase.

### Murine Colonic Crypt Isolation and Hormone Secretion Assays

Primary mice colonic crypt isolation and secretion studies were performed using an adaptation of an established method previously described 31, 32. Gut hormone secretion was expressed as a fraction of the total peptide (secreted plus intracellular) measured in each well over 2 h.

### In Vitro Mucosal Studies

Ileal or colonic mucosa from wt male mice (>15 weeks old), was voltage‐clamped at 0 mV in Ussing chambers, as described previously 33. Vectorial ion transport was measured continuously as short‐circuit current (I_sc_; μA/cm^2^) and provided an acute readout for endogenous PYY release. Once stable I_sc_ levels were achieved, vehicle or the Y_1_ receptor (Y_1_R) antagonist BIBO3304 (BIBO; 300 nM) and L‐ or D‐Arg were added to the apical or basolateral reservoirs bathing mucosae. L‐Arg (1 mM) responses were measured 15–20 min after vasoactive intestinal peptide (10 nM), an optimum secretory stimulus for revealing subsequent G_αi_‐coupled epithelial responses 33. Epithelial Y agonism results from G_αi_‐coupled attenuation of cAMP levels, with consequent sustained decreases in Cl^−^ ion secretion and I_sc_ levels 34, thus PYY (10 nM) was added after L‐Arg as a control.

### In Vivo Gut Hormone Studies

Rats were fasted overnight, before receiving o.g. of either water or 16 mmol/kg L‐Arg (n = 6–8) in the early light phase. They were immediately returned to their cages, killed by decapitation at 30 or 90 min after administration, and plasma samples were collected as previously described 29.

### Intra‐ileal Administration Studies

Intra‐ileal administration procedures were performed in anaesthetized rodents as previously described 32. Rats received an injection of either saline or 1 M L‐Arg (n = 4–5) in a volume of 2.5 ml into the upper ileum; blood samples were collected at −15, 0, 15, 30, 45 and 60 min post‐administration via the jugular cannula. Mice were injected with either saline or 1 M L‐Arg (n = 4–5) in a volume of 500 µl into the upper ileum, and were killed 30 min after administration and blood was collected.

### Gut Hormone Radioimmunoassay

We measured GLP‐1 and PYY using previously established in‐house specific and sensitive radioimmunoassays 35, 36. The GLP‐1 antibody has 100% cross‐reactivity with all amidated forms of GLP‐1, but does not cross‐react with glycine extended forms. The PYY antibody has 100% cross‐reactivity with PYY(1‐36) and PYY(3‐36). The intra‐assay coefficients of variation for GLP‐1 and PYY assays were 8.7 and 6.0%, respectively.

### Intracerebroventricular Cannulation and Administration

The intracerebroventricular (i.c.v.) injections were carried out as previously described 37. The rats recovered from surgery for 7 days before being injected with 5 µl of either vehicle saline control or 4 μM L‐Arg (n = 8–9) over 1 min using a 28‐gauge stainless steel injector in the early light phase.

### Statistical Analyses

Acute feeding studies data and area under the curve data are expressed as mean ± standard error of the mean (s.e.m.) and were analysed using one‐way analysis of variance (anova) followed by Tukey's *post hoc* test. CLAMS data were analysed using two‐way anova and a Bonferroni *post hoc* test, chronic and SDA feeding study data using multiple Student's *t‐*test, GPRC6a‐KO feeding data using two‐way anova with Sidak's *post hoc* analysis, and *in vitro* and *in vivo* gut hormone data using one‐way anova with Dunnett's and two‐way anova with Bonferroni's *post hoc* test, respectively. Mucosal data measuring the maximum changes in I_sc_, are expressed as mean ± s.e.m. per unit area (cm^2^), and single comparisons performed using Student's unpaired *t*‐test. graphpad prism software (Prism 6.03, GraphPad Software Inc, CA, USA) was used for all analyses.

## Results

### Effect of Oral L‐Arginine on Food Intake, Body Weight and Energy Expenditure in Rodents

**Figure 1:**
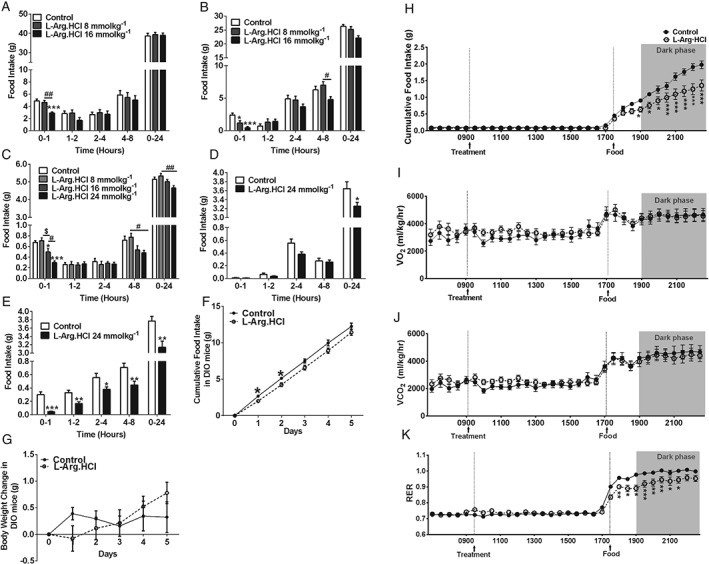
Effect of oral administration of L‐arginine (L‐Arg) on food intake, body weight and energy expenditure in rodents. Effect of oral gavage (o.g.) of control (water) and 8 and 16 mmol/kg L‐Arg on food intake in male rats (A) after an overnight fast [n = 9–10; ##P < 0.01 vs. L‐Arg (8 mmol/kg), ***P < 0.001 vs. water control] and (B) ad libitum fed at the beginning of dark phase (n = 12–16; *P < 0.05, **P < 0.01, ***P < 0.01 vs. water control, #P < 0.05 vs. 8 mmol/kg L‐Arg) at 0–1, 1–2, 2–4, 4–8 and 0–24 h after administration. (C) Effect of o.g. of control (water) and 8, 16 and 24 mmol/kg L‐Arg on food intake in male mice after an overnight fast at 0–1, 1–2, 2–4, 4–8 and 0–24 h after administration (n = 8–9; *P < 0.05, ***P < 0.001 vs. water control; #P < 0.05, ##P < 0.01 vs. 8 mmol/kg L‐Arg; $P < 0.05 vs. 16 mmol/kg L‐Arg). Effect of o.g. of control (water) or 24 mmol/kg L‐Arg in ad libitum‐fed mice during the early light phase (D) (n = 10 per group), and (E) early dark phase (n = 10 per group) at 0–1, 1–2, 2–4, 4–8, and 0–24 h after administration (*P < 0.05, **P < 0.01 ***P < 0.001 vs. control). Effect of repeated o.g. administration of L‐Arg on food intake (F) and body weight (G) in diet‐induced obese (DIO) mice. Effect of three times daily o.g. administration of control (water; black circles, solid line) or 16 mmol/kg L‐Arg (white circles, dotted line) on cumulative food intake and body weight change in DIO mice during a period of 5 days (n = 9 per group; *P < 0.05, ***P < 0.001 vs. vehicle). Effect of o.g. administration of control (water; black circles, solid line) or 24 mmol/kg L‐Arg (white circles, dotted line) on cumulative food intake (H), O_2_ consumption (I) and CO_2_ production (J) and respiratory exchange ratio (RER) (K) in mice injected at early light phase and placed in comprehensive laboratory animal monitoring system cages. The o.g. was performed at 09:00 hours and food was returned at 17:00 hours, as indicated by the dotted line. Recordings were taken over a period of 24 h and at subsequent 24‐min intervals after administration. The shaded areas represent the dark phase from 19:00 hours (n = 12 per group; *P < 0.05, **P < 0.01, ***P < 0.001 vs. water control). All data are presented as mean ± standard error of the mean.

Oral administration of L‐Arg significantly reduced food intake in both rats and mice (Figure 1). In rats, oral administration of L‐Arg suppressed food intake in fasted rats in the early light phase (Figure 1A) and in *ad libitum*‐fed rats in the early dark phase (Figure 1B). Similarly, oral administration of L‐Arg in fasted mice reduced food intake in a dose‐dependent manner (Figure 1C). Oral administration of L‐Arg in *ad libitum*‐fed mice significantly reduced cumulative food intake 24 h after administration in the light and dark phases (Figure 1D and E). These anorectic effects were not secondary to abnormal behavioural side effects in rats (Figure S1, File S1).

After observing that L‐Arg administration could result in a sustained reduction in food intake in rodents, we investigated whether this anorectic effect could be sustained chronically and reduce body weight in a diet‐induced obese (DIO) mouse model, a commonly used model of obesity. Repeated L‐Arg administration significantly reduced food intake on day 1 and day 2 in DIO mice compared with vehicle‐treated mice (Figure 1F), although this effect was insufficient to significantly change body weight over the time period studied (Figure 1G).

To investigate the effect of L‐Arg on energy expenditure, the mice were placed in CLAMS metabolic cages. Oral administration of 24 mmol/kg L‐Arg had no significant effect on VO_2_, VCO_2_ or RER in mice during the 8 h after administration. Interestingly, oral administration of 24 mmol/kg L‐Arg still reduced food intake when food was returned 8 h after administration in mice placed in CLAMS cages, showing a delayed and sustained anorectic effect (Figure 1H). Returning food did not significantly alter VO_2_ or VCO_2_ between the treatment groups (Figure 1I and J); however, the RER was significantly lower in L‐Arg‐treated mice, after the return of food (Figure 1K).

### GPRC6A is Not Required for the Anorectic Effect of L‐Arginine in Mice

**Figure 2:**
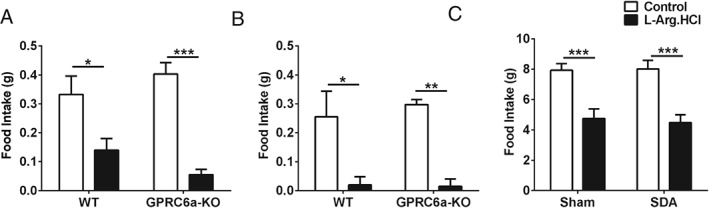
Effect of L‐arginine (L‐Arg) on food intake in G‐protein‐coupled receptor family C group 6 member A (GPRC6a)‐KO mice and rats after subdiaphragmatic vagal deafferentation (SDA) surgery. Effect of oral gavage (o.g.) administration of (A) control (water) or 16 mmol/kg L‐Arg, and (B) control (water) or 24 mmol/kg L‐Arg on 0–1‐h food intake in wildtype and GPRC6a‐KO mice with ad libitum access to food injected at the beginning of dark phase [*P < 0.05, **P < 0.01 vs. water control (n = 4, crossover)]. (C) Effect of o.g. of control (water) and 16 mmol/kg L‐Arg on 0–1‐h food intake in male rats that underwent sham or SDA surgery (n = 9–10, crossover; ***P < 0.001 vs. control). All data are presented as mean ± standard error of the mean.

Oral administration of 16 or 24 mmol/kg L‐Arg significantly reduced food intake in both wt and GPRC6a‐KO mice to a similar magnitude 0–1 h after administration, suggesting that GPRC6A is not necessary for the anorectic effect of L‐Arg (Figure 2A and B). Oral L‐Arg also improved glucose tolerance in both wt and GPRC6a‐KO mice (Figure S2, File S1).

### Anorectic Effect of L‐Arginine is Not Mediated via the Vagus

Oral gavage of 16 mmol/kg L‐Arg significantly reduced food intake in both sham‐operated and SDA‐operated rats 0–1 h after administration, suggesting the vagus is not necessary for the anorectic effect of L‐Arg (Figure 2C).

### Effect of L‐Arginine on Gut Hormone Release

**Figure 3:**
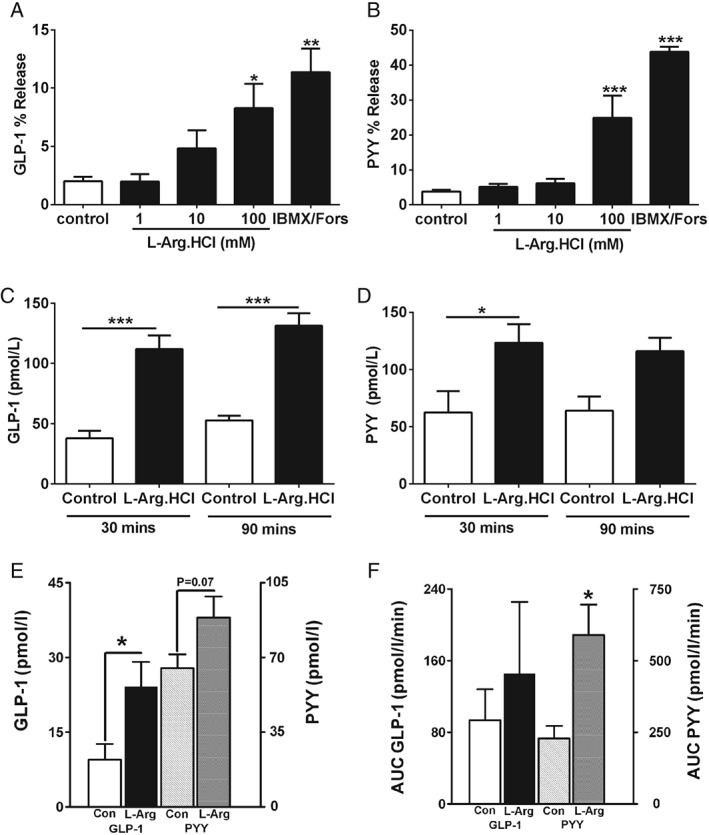
Effect of L‐arginine (L‐Arg) on gut hormone release. Effect of L‐Arg on (A) glucagon‐like peptide‐1 (GLP‐1) and (B) peptide YY (PYY) release from primary mice colonic L‐cells incubated with 1, 10 and 100 mM L‐Arg and IBMX‐forskolin mix (10 µM, each) for 2 h. The release is shown as percentages of total hormone contained for each well in the experiment (n = 9 plates from nine mice; *P < 0.05, **P < 0.01, ***P < 0.001 vs. control). Data presented as mean ± standard error of the mean (s.e.m.). Effect of oral gavage (o.g.) administration of control (water) and 16 mmol/kg L‐Arg on (C) GLP‐1, and (D) PYY in overnight fasted male rats at 30 and 90 min after administration (n = 6–8; *P < 0.05, ***P < 0.001 vs. water control, ##P < 0.01 vs. 12 mmol/kg L‐Arg). Data are presented as mean ± s.e.m. Effect of intra‐ileal administration of saline and 1 M L‐Arg on plasma GLP‐1 and PYY concentrations in overnight fasted (E) anaesthetized mice and (F) rats. Blood samples were taken from mice at 30 min, and from rats at 0, 15, 30, 45 and 60 min after administration (n = 4–5 per group; *P < 0.05 vs. control). Mice results are expressed as mean ± s.e.m. Rat results are expressed as area under the curve (AUC) mean ± s.e.m.

L‐Arginine stimulated GLP‐1 and PYY release from murine primary colonic L‐cells (Figure 3A and B). Exposure to 100 mM L‐Arg for 2 h stimulated PYY release from colonic cultures isolated from GPRC6a‐KO, although the GLP‐1 response to L‐Arg appeared to be attenuated in GPRC6a‐KO compared with wt mice (Figure S3, File S1). In keeping with observations *in vitro*, oral administration of 16 mmol/kg L‐Arg elevated plasma GLP‐1 and PYY release in rats. Plasma GLP‐1 levels were significantly elevated at 30 and 90 min after administration compared with control (Figure 3C). PYY levels were significantly elevated at 30 min after administration (Figure 3D). Furthermore, direct upper ileal administration of 1 M L‐Arg elevated plasma GLP‐1 (*P* < 0.05) and PYY (*P* = 0.07) levels in anaesthetized mice and rats (Figure 3E and F).

### Effects of L‐Arginine‐Stimulated Gut Hormone Release

**Figure 4:**
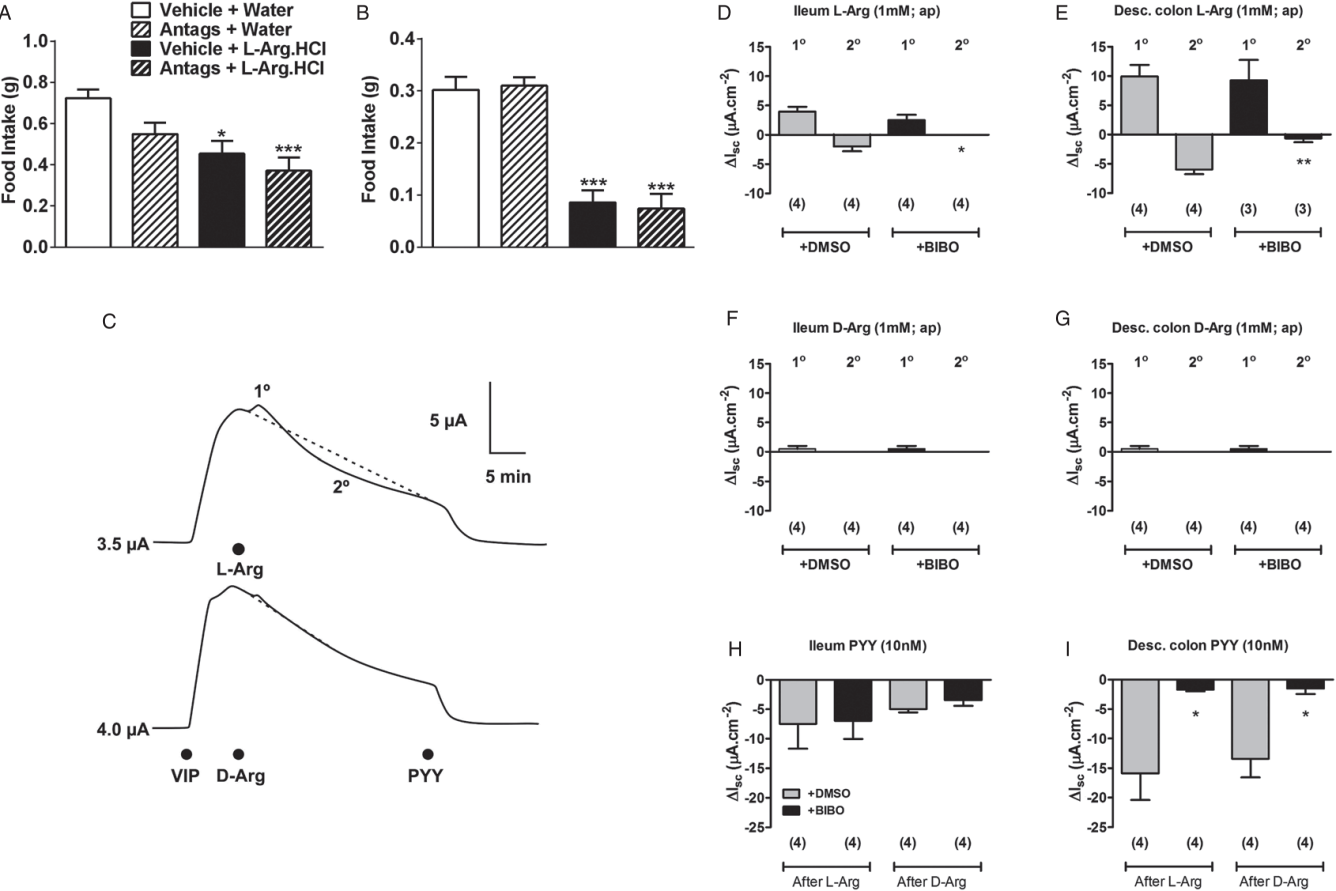
Effect of L‐arginine (L‐Arg)‐mediated gut hormone release on food intake and gut function. Effect of intraperitoneal administration of a mixture of 400 nmol/kg exendin 9‐39 and 5.26 µmol/kg BIIE0246 on the anorectic effect of oral gavage of 24 mmol/kg L‐Arg in (A) fasted mice during early light phase (n = 10) and (B) ad libitum ‐fed mice during dark phase (n = 10) in the 0–1‐h period after administration (*P < 0.05, ***P < 0.001 vs. vehicle control group). Data are presented as mean ± standard error of the mean (s.e.m.). (C) Representative recordings from mouse colon mucosa showing a biphasic I_sc_ change to apical L‐Arg (1 mM, upper) compared with minor effects to apical D‐Arg (1 mM, lower). Basolateral vasoactive intestinal peptide (10 nM) pre‐treatment increased I_sc_, and subsequent control PYY (10 nM, basolateral) anti‐secretory responses are evident. Basal I_sc_ values (in μA) are shown to the left of each trace (exposed mucosal area, 0.14 cm^2^). Responses to apical L‐Arg, D‐Arg and control PYY responses in ileum (D, F and H) and colon (E, G and I) colon are shown after either vehicle (+DMSO, 0.03%) or Y_1_R antagonist BIBO3304 (+BIBO, 300 nM). Responses are the mean ± s.e.m. from observation numbers in parenthesis. Only L‐Arg 2° I_sc_ reductions were sensitive to BIBO treatment in (D) ileum and (E) colon mucosae. Note PYY responses in the ileum (H) are attributable to Y_2_ signalling (and thus are not significantly reduced by BIBO) while Y_1_R signalling predominates in the mouse colon and is BIBO‐sensitive (I). *P < 0.05, **P < 0.01. All data are presented as mean ± s.e.m.

To investigate whether the anorectic effect of L‐Arg is mediated by increases in gut hormone levels, GLP‐1 and Y_2_ receptors were simultaneously antagonized in mice receiving 24 mmol/kg L‐Arg o.g. The GLP‐1 receptor antagonist exendin9‐39 and the Y_2_ receptor antagonist BIIE0246 were administered i.p. at doses established to block the anorectic effects of exogenous exendin‐4 and PYY, respectively (Figure S4, File S1). O.g. of 24 mmol/kg L‐Arg significantly reduced food intake, both in fasted mice in the early light phase and fed mice in the early dark phase, whether they were co‐administered saline control or a mixture of 5.26 µmol/kg BIIE0246 and 400 nmol/kg exendin9‐39 (Figure 4A and B).

In line with the hormone release measured *in vitro* and *in vivo*, mucosal I_sc_ measurements showed that L‐Arg (1 mM) altered ion transport acutely within 15–30 min, while D‐Arg was inactive (Figure 3C–G). Apical treatment of ileal and colonic mucosae with L‐Arg increased I_sc_ initially (potentially a GLP‐1‐mediated effect). It then decreased I_sc_ more slowly and, importantly, this response component was Y_1_R‐dependent and therefore most likely PYY‐mediated (Figure 4C–I).

### Effect of Central and Intraperitoneal Administration of L‐Arginine on Food Intake in Rodents

**Figure 5:**
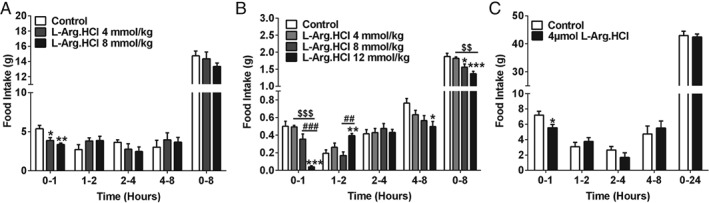
Effect of intraperitoneal (i.p.) and intracerebroventricular (i.c.v.) administration of L‐arginine (L‐Arg) on food intake in rodents. Effect of i.p. administration of (A) control (saline), 4, and 8 mmol/kg L‐Arg on food intake in fasted male rats (n = 8–9, *P < 0.05, **P < 0.01 vs. control) and (B) control (saline), 4, 8 and 12 mmol/kg L‐Arg in fasted male mice (n = 7–9; *P < 0.05, **P < 0.01, ***P < 0.001 vs. control; $$P < 0.01, $$$P < 0.001 vs. 4 mmol/kg L‐Arg; ##P < 0.01, ###P < 0.001 vs. 8 mmol/kg L‐Arg) at 0–1, 1–2, 2–4, 4–8 and 0–8 h after administration during early light phase. (C) Effect of i.c.v. administration of control (saline) and 4 µM L‐Arg on food intake in male rats following an overnight fast at 0–1, 1–2, 2–4, 4–8 and 0–24 h after administration (n = 8–9; *p < 0.05 vs. control). All data are presented as mean ± s.e.m.

The i.p. administration of 4 and 8 mmol/kg L‐Arg significantly reduced food intake in rats 0–1 h after administration compared with saline controls (Figure 5A). Similarly, in mice, 12 mmol/kg L‐Arg significantly reduced food intake 0–1 h after administration. Food intake was significantly lower in mice treated with 12 mmol/kg L‐Arg 4–8 h after administration. The cumulative food intake at 8 h post‐administration was significantly lower in both 8 and 12 mmol/kg L‐Arg groups compared with the saline control (Figure 5B). Furthermore, i.c.v. administration of L‐Arg significantly reduced food intake in rats at 0–1 h post‐administration only, but had no effect on 0–24 h cumulative food intake (Figure 5C).

## Discussion

We investigated the anorectic properties of L‐Arg in rodents and the potential mechanisms by which these effects are mediated. Our data show that L‐Arg reduces food intake in both mice and rats without causing behavioural side effects, but does not affect energy expenditure in mice at the dose investigated. Repeated L‐Arg administration reduced cumulative food intake on days 1 and 2 in DIO mice, but without significant effect on body weight over the time studied. The anorectic effects of L‐Arg were not dependent on the amino acid sensing receptor GPRC6A or on vagal signalling. L‐Arg significantly stimulated GLP‐1 and PYY release acutely *in vitro* and *in vivo*; however, its anorectic effects appear unlikely to be mediated by changes in these gut hormones.

The anorectic effect of L‐Arg has been previously shown in a rat model. Jordi et al. 23 showed a significant reduction in food intake after o.g. of 6.7 mmol/kg L‐Arg in rats. In our initial dose finding studies, doses of 4 and 6 mmol/kg of the non‐salt L‐Arg solution suppressed food intake in rats; however, equivalent doses of the L‐Arg·HCl salt had no effect on food intake and a dose of 8 mmol/kg or higher was required to significantly reduce food intake in rats. This suggests that the non‐salt L‐Arg may influence food intake at least in part because of its basicity. To avoid any possible pH‐dependent effect, the neutral L‐Arg·HCl salt was used in all of our reported experiments. While it is possible that L‐Arg·HCl solution has non‐specific osmotic effects on food intake, pilot studies using isomolar concentrations of sodium chloride showed no effect of this concentration of sodium choloride on food intake, and found that L‐Arg reduced food intake compared with iso‐osmotic controls (data not shown).

We found that acute administration of L‐Arg had no significant effect on VO_2_ and VCO_2_ in mice; however, the study showed that o.g. of 24 mmol/kg L‐Arg significantly reduced food intake in mice when food was returned 8 h later. This shows a prolonged anorectic effect that is not necessarily observable if food is returned immediately. It also suggests L‐Arg can have long‐term effects on food intake when food is not immediately available after administration, which may be exploitable by weight‐loss promoting agents. Furthermore, RER was significantly lower in the L‐Arg‐treated cohort once food was returned. This effect probably reflects the significantly lower food intake in the L‐Arg‐treated rodents. Different experimental conditions may alter the pharmacokinetic profile of L‐Arg, and further work is required to determine whether such long‐term effects occur in other contexts. It is also possible that the effects observed reflect the action of L‐Arg metabolites or other molecules of which it is a precursor, such as nitric oxide and glutamate; however, simple availability of L‐Arg does not regulate the levels of many of these downstream agents.

Chronic L‐Arg supplementation in mice on a low protein diet has been reported to reduce epididymal fat, while increasing food intake 38. L‐Arg may facilitate increased protein synthesis in animals deficient in protein, but its effects might be expected to be very different in rodents with a normal protein intake. We found repeated administration of L‐Arg reduced cumulative food intake but did not significantly influence weight gain in DIO mice. A longer period of administration and perhaps a higher dose might result in a significant effect on body weight. Our data do not exclude the possibility of small changes to body weight that we were unable to detect in response to chronic L‐Arg administration. It is also possible that L‐Arg promotes the absorption of other nutrients or causes small decreases in energy expenditure that were not detectable by the CLAMS, which would explain the lack of difference in body weight. Further work is required to establish the chronic effects of L‐Arg administration on energy homeostasis.

L‐Arg can influence hormone release from other endocrine tissues including the pancreas 17 and the pituitary gland 22. We therefore investigated the role of anorectic gut hormones in mediating the effect of L‐Arg on food intake. L‐Arg stimulated the release of GLP‐1 and PYY *in vitro* and *in vivo*. Our data suggested that the anorectic effects of L‐Arg are not mediated via gut hormone release, although a potential role for other gut hormones cannot be excluded. Mucosal studies, however, complemented the hormonal release we observed and indicated that L‐Arg caused acute endogenous PYY release with consequent rapid inhibition of local epithelial ion transport that was Y_1_R‐mediated. This mechanism is similar to that described for other amino acids acting via the CaSR in mouse colon 39. PYY and GLP‐1 inhibit gastric emptying, the former most likely acting via Y1 and Y2 receptors 40; PYY(1‐36) binds to both receptors with similar affinity, while the truncated form which reduces appetite has higher affinity for the Y2 receptor. Furthermore, L‐Arg reduces gastric emptying in humans, apparently via changes in basal levels of nitric oxide 41, 42. In addition, both GLP‐1 and PYY inhibit gastric motility in rats 43, 44. Increased plasma GLP‐1 and PYY after L‐Arg administration may slow gastric emptying acutely, but our evidence suggests that these rapid changes in hormone levels may not be responsible for the subsequent reduction in food intake, which was not blocked by antagonising either GLP‐1 or PYY‐Y2 receptors. Both PYY(1‐36) and PYY(3‐36), however, probably influence upper gastrointestinal transit, including gastric emptying, in mice 40, and it is therefore possible that the effects of L‐Arg on PYY(1‐36) release alter gastric emptying via the Y1 receptor sufficiently to account for some of the observed anorectic effect in mice.

Basic amino acids including L‐Arg are potent activators of GPRC6A 19, which is highly expressed in the gastrointestinal tract and is involved in a number of important physiological pathways 45. The effects of L‐Arg were examined in mice lacking the GPRC6A at both 24 mmol/kg, which was previously shown to reduce food intake in mice, and at a lower dose of 16 mmol/kg, in case the effects of higher doses were mediated by different, perhaps non‐physiological mechanisms; however, at both doses, oral administration of L‐Arg significantly reduced food intake in GPRC6a‐KO mice. Previously, small interfering RNA‐induced depletion of endogenous GPRC6A has been shown to abolish L‐ornithine‐stimulated GLP‐1 release from GLUTag cells 21. GPRC6A ablation did not appear to block L‐Arg‐induced GLP‐1 and PYY release from a primary cultured murine colonic epithelium, although the effect on GLP‐1 release was attenuated. These data suggest that GPRC6A is not necessary for the anorectic effects of L‐Arg, and that it plays at most a minor role in its effects on gut hormone release. L‐Arg also activates both T1R1‐T1R3 and CaSR receptors, albeit to a lesser extent than GPRC6A 7. The involvement of these receptors cannot be ruled out. L‐Arg‐induced GLP‐1 and PYY release from isolated rat small intestinal loops was attenuated by a CaSR antagonist, suggesting it is in part mediated by CaSR 15.

Other mechanisms may be involved in mediating the effects of L‐Arg. Evidence suggests L‐Arg stimulates the release of insulin from pancreatic β‐cells by causing membrane depolarization, and that this effect is not mediated by calcium or ATP‐sensitive potassium channels, but as a consequence of electrogenic transport of L‐Arg into the β‐cell via specific amino acid transporters 46. Amino acid transporter systems may also be involved in amino acid sensing in the gut 14. *In vitro* studies suggest that L‐cells exhibit action potential‐driven calcium influx in response to nutrients including amino acids, leading to acute hormone release. The sodium‐coupled neutral amino acid transporter 2 (SNAT2) has been implicated in nutrient sensing and gut hormone release. SNAT2 acts as a secondary active transporter by coupling the transfer of amino acids against their concentration gradient to the simultaneous inward movement of sodium ions down its electrochemical gradient. This sodium‐dependent transport mechanism has been shown to increase intracellular calcium levels and consequently to stimulate the release of gut hormones 47. Notably, L‐glutamine has been shown to stimulate GLP‐1 release from intestinal L‐cells via a SNAT2‐mediated mechanism 14. In addition, the CaSR has been shown to mediate the pharmacological effects of specific amino acids on gut hormone release from cell lines and *ex‐vivo* tissue 15, 48.

There is evidence that the vagus nerve responds to nutrient load and is involved in protein‐induced satiety 3. Proteins and amino acids activate neurons within the nucleus of the solitary tract via visceral vagus mediated signals. In addition, GLP‐1, Y_2_ and CCK receptors are expressed on vagal afferents which are proposed to play a key role in gut‐brain mediated responses in satiety and food intake regulation 49. We examined the effect of L‐Arg on food intake in rats that had undergone SDA surgery and observed no difference on the effects of L‐Arg on food intake, in accord with previous work suggesting the anorectic effect of L‐Arg is not vagally‐mediated 23.

The i.p. or i.c.v. administration of L‐Arg significantly reduced food intake in rats. These findings raise the possibility that a post‐absorptive mechanism may be involved in mediating the anorectic effects of L‐Arg. Jordi et al. 23 suggested that the anorectic effect of L‐Arg is mediated centrally via the area postrema (AP) in the brain stem. Oral administration of L‐Arg solution increased c‐fos positive cells in AP, and the anorectic effect of L‐Arg was abolished in animals that had undergone AP lesioning surgery, although, as mentioned earlier, these studies were performed using the non‐salt, basic L‐Arg solution. We have previously reported an increase in c‐fos‐positive cells in the AP after oral L‐cysteine administration, suggesting that there may be similar mechanisms by which amino acids influence food intake 29. Branched‐chain amino acids have also been shown to reduce food intake in rodents centrally 50. Recent studies suggest that a member of the soluble carrier family of proteins, SLC38A9, may play a role in central L‐Arg sensing via mammalian target of rapamycin complex 1‐dependent mechanisms 51. Further studies are required to investigate the putative role of post‐absorptive mechanisms in the anorectic effects of L‐Arg.

In summary, our data further demonstrate the anorectic properties of L‐Arg and explore the potential mechanisms involved. The doses of L‐Arg administered orally were pharmacological, with the amounts administered acutely being of a similar order of magnitude to the levels that a rodent would consume daily on a 45% high protein diet; however, the present results may also represent pharmacological activation of a physiological nutrient‐sensing system. The present chronic administration study suggests that L‐Arg may not reduce body weight after repeated dosing, but further work is required to establish the mechanisms involved in mediating the anorectic effects of L‐Arg and to explore whether altering the dose and timing of chronic administration might result in significant effects on body weight, and thus suggest therapeutic potential in obesity.

## Conflict of Interest

This paper presents independent research funded by Biotechnology and Biological Sciences Research Council (BBSRC), MRC and Society for Endocrinology. The views expressed are those of the author(s) and not necessarily those of the funders. The Section of Investigative Medicine is funded by grants from the Medical research council (MRC), Biotechnology and Biological Sciences Research Council (BBSRC), NIHR, an Integrative Mammalian Biology (IMB) Capacity Building Award, an FP7‐HEALTH‐2009‐241592 EuroCHIP grant and is supported by the NIHR Biomedical Research Centre Funding Scheme. A. Alamshah was funded by Innovate UK (Technology Strategy Board), A. K. M. and J. K‐J. were funded by BBSRC, E. S. was funded by National Centre for the Replacement Refinement & Reduction of Animals in Research (NC3Rs) and A. Amin and K. B. were funded by MRC. The authors declare no conflict of interest.

A. Alamshah designed and performed the experiments, analysed the data and wrote the manuscript. A. K. M. designed and performed the experiments and analysed the data. E. S., J. K‐J., H. C. O. and I. R. T. performed experiments. A. A., A. M., K. B., R. F., G. H., M. N. and W. C. assisted with experiments. A. L. and H. M. C. designed experiments, provided material support and helped edit the manuscript. S. R. B. provided material and technical support. K. G. M. wrote the manuscript and was responsible for study concept, design and analysis.

## Supporting information


**File S1.** L‐Arginine promotes gut hormone release and reduces food intake in rodents.
**Table S1.** Feeding studies conditions. Table details the experimental conditions including species, phase of the study, feeding state of the cohort, route of administration and the dose of L‐Arg used for each of the feeding studies described in the manuscript. The Feeding study (figure) column refers to the figure number showing the results for each study in the main manuscript. *All the doses presented in mmol/kg with exception of the ICV study where the dose is given in µmol.
**Figure S1.** The effects of oral administration of L‐Arg on behaviour in fasted rats during the light phase. The effect of OG of water (control) or 16 mmol/kg L‐Arg in overnight fasted male rats on feeding, locomotion, grooming, head down, pica and resting behaviours compared to control group. Data represented as median (interquartile range) for each observation. n = 12–13.
**Figure S2.** The effect of L‐Arg on glucose homeostasis in GPRC6a‐KO mice. Glucose tolerance test (GTT) in female WT and GPRC6a‐KO mice (A) and the area under the curve for each treatment (B). Mice were fasted overnight and received an intraperitoneal injection of 20% glucose solution (2 g/kg body weight) followed by an immediate OG of 4 mmol/kg L‐Arg. Data is presented as mean ± SEM. n = 6 per group. *P < 0.05, ***P < 0.001 WT‐L‐Arg vs. WT‐L‐saline; ###P < 0.001 vs. GPRC6a‐KO‐L‐Arg vs. GPRC6a‐WT.
**Figure S3.** The effect of GPRC6A on GLP‐1 and PYY release from primary murine colonic epithelium. The effect of L‐Arg on GLP‐1 (A) and PYY (B) release from WT and GPRC6A‐KO primary mice colonic L‐cells incubated with 100 mM L‐Arg for 2 h. The release is shown as percentage of total hormone contained for each well in the experiment. Data is presented as mean ± SEM. n = 6 plates, from 6 mice. *p < 0.05, ***p < 0.001 vs. control.
**Figure S4.** The effect of GLP‐1 and Y2 receptors antagonism on the effects of exogenous exendin‐4 and PYY on food intake in mice. The effect of IP administration of 400 nmol/kg exendin 9‐39 on the anorectic effect of 1 nmol/kg exogenous exendin‐4 in fasted mice at 0–1 h post administration (n = 10) (A). The effect of IP administration of 5.26 µmol/kg BIIE0246 on the anorectic effect of 25 nmol/kg PYY(3‐36) in fasted mice at 0–1 h post administration (n = 10) (B). Data is presented as mean ± SEM. (A): **P < 0.01 vs. saline control, ###P < 0.001 vs. exendin 9‐39, $$P < 0.01 vs. exendin‐4; (B): ***P < 0.001 vs vehicle control, ###P < 0.001 vs. BIIE0246, $$P < 0.01 vs. PYY(3‐36).

## References

[1] Poppitt SD , McCormack D , Buffenstein R . Short‐term effects of macronutrient preloads on appetite and energy intake in lean women. Physiol Behav 1998; 64: 279–285. 9748094 10.1016/s0031-9384(98)00061-4

[2] Westerterp‐Plantenga MS , Lejeune MP , Nijs I , van Ooijen M , Kovacs EM . High protein intake sustains weight maintenance after body weight loss in humans. Int J Obes Relat Metab Disord 2004; 28: 57–64. 14710168 10.1038/sj.ijo.0802461

[3] Fromentin G , Darcel N , Chaumontet C , Marsset‐Baglieri A , Nadkarni N , Tome D . Peripheral and central mechanisms involved in the control of food intake by dietary amino acids and proteins. Nutr Res Rev 2012; 25: 29–39. 22643031 10.1017/S0954422411000175

[4] Halton TL , Hu FB . The effects of high protein diets on thermogenesis, satiety and weight loss: a critical review. J Am Coll Nutr 2004; 23: 373–385. 15466943 10.1080/07315724.2004.10719381

[5] Koehnle TJ , Russell MC , Gietzen DW . Rats rapidly reject diets deficient in essential amino acids. J Nutr 2003; 133: 2331–2335. 12840202 10.1093/jn/133.7.2331

[6] Veldhorst M , Smeets A , Soenen S et al. Protein‐induced satiety: effects and mechanisms of different proteins. Physiol Behav 2008; 94: 300–307. 18282589 10.1016/j.physbeh.2008.01.003

[7] Wellendorph P , Johansen LD , Brauner‐Osborne H . Molecular pharmacology of promiscuous seven transmembrane receptors sensing organic nutrients. Mol Pharmacol 2009; 76: 453–465. 19487246 10.1124/mol.109.055244

[8] Potier M , Darcel N , Tome D . Protein, amino acids and the control of food intake. Curr Opin Clin Nutr Metab Care 2009; 12: 54–58. 19057188 10.1097/MCO.0b013e32831b9e01

[9] Wang Y , Chandra R , Samsa LA et al. Amino acids stimulate cholecystokinin release through the Ca2 + ‐sensing receptor. Am J Physiol Gastrointest Liver Physiol 2011; 300: G528–G537. 21183662 10.1152/ajpgi.00387.2010 PMC3074989

[10] Spreckley E , Murphy KG . The L‐Cell in nutritional sensing and the regulation of appetite. Front Nutr 2015; 2: 23. 26258126 10.3389/fnut.2015.00023 PMC4507148

[11] Batterham RL , Heffron H , Kapoor S et al. Critical role for peptide YY in protein‐mediated satiation and body‐weight regulation. Cell Metab 2006; 4: 223–233. 16950139 10.1016/j.cmet.2006.08.001

[12] van der Klaauw AA , Keogh JM , Henning E et al. High protein intake stimulates postprandial GLP1 and PYY release. Obesity 2013; 21: 1602–1607. 23666746 10.1002/oby.20154 PMC6548554

[13] Berthoud HR . Vagal and hormonal gut‐brain communication: from satiation to satisfaction. Neurogastroenterol Motil 2008; 20(Suppl. 1): 64–72. 18402643 10.1111/j.1365-2982.2008.01104.x PMC3617963

[14] Tolhurst G , Zheng Y , Parker HE , Habib AM , Reimann F , Gribble FM . Glutamine triggers and potentiates glucagon‐like peptide‐1 secretion by raising cytosolic Ca2+ and cAMP. Endocrinology 2011; 152: 405–413. 21209017 10.1210/en.2010-0956 PMC3140224

[15] Mace OJ , Schindler M , Patel S . The regulation of K‐ and L‐cell activity by GLUT2 and the calcium‐sensing receptor CasR in rat small intestine. J Physiol 2012; 590(Pt 12): 2917–2936. 22495587 10.1113/jphysiol.2011.223800 PMC3448156

[16] Wu G , Morris SM Jr . Arginine metabolism: nitric oxide and beyond. Biochem J 1998; 336(Pt 1): 1–17. 9806879 10.1042/bj3360001 PMC1219836

[17] Adeghate E , Ponery AS , El‐Sharkawy T , Parvez H . L‐arginine stimulates insulin secretion from the pancreas of normal and diabetic rats. Amino Acids 2001; 21: 205–209. 11665817 10.1007/s007260170028

[18] Clemmensen C , Smajilovic S , Smith EP et al. Oral L‐arginine stimulates GLP‐1 secretion to improve glucose tolerance in male mice. Endocrinology 2013; 154: 3978–3983. 23959939 10.1210/en.2013-1529 PMC3800753

[19] Wellendorph P , Hansen KB , Balsgaard A , Greenwood JR , Egebjerg J , Brauner‐Osborne H . Deorphanization of GPRC6A: a promiscuous L‐alpha‐amino acid receptor with preference for basic amino acids. Mol Pharmacol 2005; 67: 589–597. 15576628 10.1124/mol.104.007559

[20] Pi M , Wu Y , Lenchik NI , Gerling I , Quarles LD . GPRC6A mediates the effects of L‐arginine on insulin secretion in mouse pancreatic islets. Endocrinology 2012; 153: 4608–4615. 22872579 10.1210/en.2012-1301 PMC3512028

[21] Oya M , Kitaguchi T , Pais R , Reimann F , Gribble F , Tsuboi T . The G protein‐coupled receptor family C group 6 subtype A (GPRC6A) receptor is involved in amino acid‐induced glucagon‐like peptide‐1 secretion from GLUTag cells. J Biol Chem 2013; 288: 4513–4521. 23269670 10.1074/jbc.M112.402677 PMC3576058

[22] Villalobos C , Nunez L , Garcia‐Sancho J . Mechanisms for stimulation of rat anterior pituitary cells by arginine and other amino acids. J Physiol 1997; 502 (Pt 2): 421–431. 9263921 10.1111/j.1469-7793.1997.421bk.x PMC1159560

[23] Jordi J , Herzog B , Camargo SM , Boyle CN , Lutz TA , Verrey F . Specific amino acids inhibit food intake via the area postrema or vagal afferents. J Physiol 2013; 591(Pt 22): 5611–5621. 23897232 10.1113/jphysiol.2013.258947 PMC3853499

[24] Kinsey‐Jones JS , Alamshah A , McGavigan AK et al. GPRC6a is not required for the effects of a high‐protein diet on body weight in mice. Obesity 2015; 23: 1194–1200. 25958858 10.1002/oby.21083 PMC4692088

[25] Pi M , Chen L , Huang MZ et al. GPRC6A null mice exhibit osteopenia, feminization and metabolic syndrome. PLoS One 2008; 3: e3858. 19050760 10.1371/journal.pone.0003858 PMC2585477

[26] Wellendorph P , Johansen LD , Jensen AA et al. No evidence for a bone phenotype in GPRC6A knockout mice under normal physiological conditions. J Mol Endocrinol 2009; 42: 215–223. 19103720 10.1677/JME-08-0149

[27] Smajilovic S , Clemmensen C , Johansen LD et al. The L‐alpha‐amino acid receptor GPRC6A is expressed in the islets of Langerhans but is not involved in L‐arginine‐induced insulin release. Amino Acids 2013; 44: 383–390. 22714012 10.1007/s00726-012-1341-8

[28] Semjonous NM , Smith KL , Parkinson JR et al. Coordinated changes in energy intake and expenditure following hypothalamic administration of neuropeptides involved in energy balance. Int J Obes 2009; 33: 775–785. 10.1038/ijo.2009.96 PMC2711051 19488048

[29] McGavigan AK , O'Hara HC , Amin A et al. l‐cysteine suppresses ghrelin and reduces appetite in rodents and humans. Int J Obes 2015; 39: 447–455. 10.1038/ijo.2014.172 PMC4276721 25219528

[30] Arnold M , Mura A , Langhans W , Geary N . Gut vagal afferents are not necessary for the eating‐stimulatory effect of intraperitoneally injected ghrelin in the rat. J Neurosci 2006; 26: 11052–11060. 17065447 10.1523/JNEUROSCI.2606-06.2006 PMC6674670

[31] Reimann F , Habib AM , Tolhurst G , Parker HE , Rogers GJ , Gribble FM . Glucose sensing in L cells: a primary cell study. Cell Metab 2008; 8: 532–539. 19041768 10.1016/j.cmet.2008.11.002 PMC2697331

[32] Psichas A , Sleeth ML , Murphy KG et al. The short chain fatty acid propionate stimulates GLP‐1 and PYY secretion via free fatty acid receptor 2 in rodents. Int J Obes 2015; 39: 424–429. 10.1038/ijo.2014.153 PMC4356745 25109781

[33] Cox HM , Pollock EL , Tough IR , Herzog H . Multiple Y receptors mediate pancreatic polypeptide responses in mouse colon mucosa. Peptides 2001; 22: 445–452. 11287100 10.1016/s0196-9781(01)00355-2

[34] Cox HM , Cuthbert AW , Hakanson R , Wahlestedt C . The effect of neuropeptide Y and peptide YY on electrogenic ion transport in rat intestinal epithelia. J Physiol 1988; 398: 65–80. 3392683 10.1113/jphysiol.1988.sp017029 PMC1191759

[35] Kreymann B , Williams G , Ghatei MA , Bloom SR . Glucagon‐like peptide‐1 7‐36: a physiological incretin in man. Lancet 1987; 2: 1300–1304. 2890903 10.1016/s0140-6736(87)91194-9

[36] Adrian TE , Ferri GL , Bacarese‐Hamilton AJ , Fuessl HS , Polak JM , Bloom SR . Human distribution and release of a putative new gut hormone, peptide YY. Gastroenterology 1985; 89: 1070–1077. 3840109 10.1016/0016-5085(85)90211-2

[37] McGowan BM , Stanley SA , Smith KL et al. Central relaxin‐3 administration causes hyperphagia in male Wistar rats. Endocrinology 2005; 146: 3295–3300. 15845619 10.1210/en.2004-1532

[38] Clemmensen C , Madsen AN , Smajilovic S , Holst B , Brauner‐Osborne H . L‐Arginine improves multiple physiological parameters in mice exposed to diet‐induced metabolic disturbances. Amino acids. 2012; 43: 1265–1275. 22200933 10.1007/s00726-011-1199-1

[39] Joshi S , Tough IR , Cox HM . Endogenous PYY and GLP‐1 mediate l‐glutamine responses in intestinal mucosa. Br J Pharmacol 2013; 170: 1092–1101. 23992397 10.1111/bph.12352 PMC3902494

[40] Tough IR , Forbes S , Tolhurst R et al. Endogenous peptide YY and neuropeptide Y inhibit colonic ion transport, contractility and transit differentially via Y(1) and Y(2) receptors. Br J Pharmacol 2011; 164: 471–484. 21457230 10.1111/j.1476-5381.2011.01401.x PMC3188896

[41] Konturek JW , Thor P , Domschke W . Effects of nitric oxide on antral motility and gastric emptying in humans. Eur J Gastroenterol Hepatol 1995; 7: 97–102. 7712314

[42] Fiorucci S , Distrutti E , Quintieri A et al. L‐arginine/nitric oxide pathway modulates gastric motility and gallbladder emptying induced by erythromycin and liquid meal in humans. Dig Dis Sci 1995; 40: 1365–1371. 7781462 10.1007/BF02065553

[43] Chen CH , Rogers RC , Stephens RL Jr . Intracisternal injection of peptide YY inhibits gastric emptying in rats. Regul Pept 1996; 61: 95–98. 8852810 10.1016/0167-0115(95)00143-3

[44] Imeryuz N , Yegen BC , Bozkurt A , Coskun T , Villanueva‐Penacarrillo ML , Ulusoy NB . Glucagon‐like peptide‐1 inhibits gastric emptying via vagal afferent‐mediated central mechanisms. Am J Physiol 1997; 273(Pt 1): G920–G927. 9357836 10.1152/ajpgi.1997.273.4.G920

[45] Clemmensen C , Smajilovic S , Wellendorph P , Brauner‐Osborne H . The GPCR, class C, group 6, subtype A (GPRC6A) receptor: from cloning to physiological function. Br J Pharmacol 2014; 171: 1129–1141. 24032653 10.1111/bph.12365 PMC3952793

[46] Smith PA , Sakura H , Coles B , Gummerson N , Proks P , Ashcroft FM . Electrogenic arginine transport mediates stimulus‐secretion coupling in mouse pancreatic beta‐cells. J Physiol 1997; 499(Pt 3): 625–635. 9130159 10.1113/jphysiol.1997.sp021955 PMC1159281

[47] Young SH , Rey O , Sternini C , Rozengurt E . Amino acid sensing by enteroendocrine STC‐1 cells: role of the Na + ‐coupled neutral amino acid transporter 2. Am J Physiol Cell Physiol 2010; 298: C1401–C1413. 20219951 10.1152/ajpcell.00518.2009 PMC2889636

[48] Daly K , Al‐Rammahi M , Moran A , Marcello M , Ninomiya Y , Shirazi‐Beechey SP . Sensing of amino acids by the gut‐expressed taste receptor T1R1‐T1R3 stimulates CCK secretion. Am J Physiol Gastrointest Liver Physiol 2013; 304: G271–G282. 23203156 10.1152/ajpgi.00074.2012 PMC3566511

[49] Tome D , Schwarz J , Darcel N , Fromentin G . Protein, amino acids, vagus nerve signaling, and the brain. Am J Clin Nutr 2009; 90: 838S–843S. 19640948 10.3945/ajcn.2009.27462W

[50] Cota D , Proulx K , Smith KA et al. Hypothalamic mTOR signaling regulates food intake. Science 2006; 312: 927–930. 16690869 10.1126/science.1124147

[51] Wang S , Tsun ZY , Wolfson RL et al. Metabolism. Lysosomal amino acid transporter SLC38A9 signals arginine sufficiency to mTORC1. Science 2015; 347: 188–194. 25567906 10.1126/science.1257132 PMC4295826

