# Supplementary material for: L‐arginine promotes gut hormone release and reduces food intake in rodents
Source: Diabetes Obes Metab. 2016 Apr 1;18(5):508–18. doi: 10.1111/dom.12644 (PMC4982043; doi:10.1111/dom.12644)
Supplement: Supplementary file 1 — File S1. L‐Arginine promotes gut hormone release and reduces food intake in rodents. Table S1. Feeding studies conditions. Table details the experimental conditions including species, phase of the study, feeding state of the cohort, route of administration and the dose of L‐Arg used for each of the feeding studies described in the manuscript. The Feeding study (figure) column refers to the figure number showing the results for each study in the main manuscript. *All the doses presented in mmol/kg with exception of the ICV study where the dose is given in µmol. Figure S1. The effects of oral administration of L‐Arg on behaviour in fasted rats during the light phase. The effect of OG of water (control) or 16 mmol/kg L‐Arg in overnight fasted male rats on feeding, locomotion, grooming, head down, pica and resting behaviours compared to control group. Data represented as median (interquartile range) for each observation. n = 12–13. Figure S2. The effect of L‐Arg on glucose homeostasis in GPRC6a‐KO mice. Glucose tolerance test (GTT) in female WT and GPRC6a‐KO mice (A) and the area under the curve for each treatment (B). Mice were fasted overnight and received an intraperitoneal injection of 20% glucose solution (2 g/kg body weight) followed by an immediate OG of 4 mmol/kg L‐Arg. Data is presented as mean ± SEM. n = 6 per group. *P < 0.05, ***P < 0.001 WT‐L‐Arg vs. WT‐L‐saline; ###P < 0.001 vs. GPRC6a‐KO‐L‐Arg vs. GPRC6a‐WT. Figure S3. The effect of GPRC6A on GLP‐1 and PYY release from primary murine colonic epithelium. The effect of L‐Arg on GLP‐1 (A) and PYY (B) release from WT and GPRC6A‐KO primary mice colonic L‐cells incubated with 100 mM L‐Arg for 2 h. The release is shown as percentage of total hormone contained for each well in the experiment. Data is presented as mean ± SEM. n = 6 plates, from 6 mice. *p < 0.05, ***p < 0.001 vs. control. Figure S4. The effect of GLP‐1 and Y2 receptors antagonism on the effects of exogenous exendin‐4 and PYY on food intake in mice [file DOM-18-508-s001.docx]

**Supplemental Data (Alamshah et al. 2016)**

**Supplementary methods**

*The effect of OG administration of L-Arg.HCl on behaviour in rats*

Rats were fasted for 16h overnight and were subsequently orally gavaged with either water or 16mmolkg^-1^ L-Arg. Animals were then returned to their cages and observed by a researcher blinded to the experimental treatment. Each animal was observed for 5s every 15s for a total period of 5min. The observations were carried out during the hour following administration. Animals were monitored for twelve distinct behavioural observations: feeding, drinking, rearing, locomotion, grooming, pica, bed-making, head-down/hunched, sleeping, tremors, climbing and stationary. These observations were subsequently sub-categorised into six behaviours: feeding (feeding or drinking), locomotion (rearing, locomotion, bed-making and climbing), head-down (head-down, hunched and tremors), grooming, pica and resting (stationary or sleeping) ([1](#_ENREF_1)).

*The effect of L-Arg on glucose homeostasis in GPRC6-a-KO mice*

Six to eight week old individually housed female WT and GPRC6a-KO mice were fasted for 16 hours overnight before an intraperitoneal injection of 20% glucose (2 g/kg) followed by an immediate oral gavage of 4 mmolkg^-1^ L-Arg, a similar dose to that previously demonstrated to improve glucose tolerance in mice when orally administered ([2](#_ENREF_2)). Samples for blood glucose were taken from the tail vein immediately before glucose injection (t = 0) and at 15, 30, 60, 90 and 120 minutes following administration and were measured using a 65 glucometer (CONTOUR meter and test strips, Bayer, Berkshire, UK).

*The role of GPRC6A in mediating L-Arg.HCl-induced gut hormone release in vitro*

The WT and GPRC6A-KO primary mice colonic L-cells were isolated and prepared for secretion experiments as previously described. Secretion experiments were performed using 100mM L-Arg. The control for these experiments was secretion buffer that was osmolarity-matched to 100mM L-Arg osmolarity to account for any possible osmolarity-induced effects on gut hormone release. The osmolarity of the L-Arg was measured using an osmometer (osmomat 030, Gonotec, Berlin, Germany) and control treatment osmolarity was matched using 3X concentrated secretion buffer. GLP-1 and PYY levels were measured using specific RIAs and secretion data was represented as percentage of total hormone.

*The effect of specific doses of GLP-1 and Y2 receptor antagonists on the effect of exogenous exendin-4 and PYY on food intake in mice*

Mice were fasted overnight and subsequently received an IP injection of saline or 400nmolkg^-1^ of exendin9-39 (a dose previously shown to block the effects of GLP-1 on food intake) ([3](#_ENREF_3)). This was followed by an immediate IP injection of saline or 1nmolkg^-1^ of exendin-4. Mice were returned to their cages with a pre-weighed amount of food provided, and food intake measured at 1h following administration.

This protocol was also used in a separate study to investigate the ability of BIIE0246 to inhibit the anorectic effect of exogenous PYY(3-36). The ability of BIIE0246 to block the anorectic effects of 25nmolkg^-1^ of PYY(3-36) at a previously established anorectic dose of 5.26μmolkg^-1^ ([4](#_ENREF_4)) was tested.

*Subdiaphragmatic vagal deafferentation (SDA) surgery*

Rats were adapted to a nutritionally complete liquid diet (Nestlé Nutrition, Resource Energy, 1.5 kcal ml−1) for 3 days before undergoing SDA or sham surgery. The surgery involved the left intracranial rhizotomy and transection of the dorsal subdiaphragmatic trunk of the vagus, which resulted in 50% deafferentation and complete subdiaphragmatic vagal deafferentation. The identical procedures were repeated for the sham operated animals without the lesioning of the vagal rootlets or trunk. Lesion verification was carried out at the end of the experimental series microscopically. Animals received liquid diet for 2 days and then a semiliquid diet for 4 days, and were allowed 10 days to fully recover from the surgery before the commencement of the studies.

*Murine colonic crypt isolation*

Male C57BL/6 mice between 6 and 8 weeks of age were sacrificed by cervical dislocation. The colon was dissected and collected in ice cold Leibovitz-15 (L-15) medium (PAA, UK), opened, rinsed in L-15 and chopped into 1-2 mm pieces. Tissue was then digested in 0.4 mg/ml collagenase XI (Sigma, Poole, UK) in Dulbecco's Modified Eagle Medium (DMEM) containing 10% foetal bovine serum, 100 U/ml penicillin, and 0.1 mg/ml streptomycin) for an initial 10 minute period. The supernatant was collected and the tissue was digested in fresh DMEM media containing collagenase for 15 minutes. This process was then repeated twice more. The supernatant was centrifuged at 500g for 5 minutes and the pellet resuspended in DMEM. Cell suspensions were filtered through a sterile nylon mesh (pore size 250 μM). Filtered cell suspensions were aliquoted on 24 well plates coated with 1% matrigel (BD Bioscience, Oxford, UK). Plates were incubated overnight at 37°C, 5% CO2/95% O2 prior to secretion experiments.

*Secretion experiments*

Secretion assays were performed on primary cultures 24 hours after plating and incubation. All treatments were made up in secretion buffer (4.5 mm KCl, 138 mm NaCl, 4.2 mm NaHCO_3_, 1.2 mm NaH_2_PO_4_, 2.6 mm CaCl_2_, 1.2 mmMgCl_2_, and 10 mm HEPES, adjusted to pH 7.4 with NaOH and supplemented with 0.1% fatty acid-free bovine albumin serum (BSA; Sigma)). Media was removed and plates were washed twice with secretion buffer. Experimental controls were osmolarity-matched when high concentrations of salt-based treatments were tested using 3X concentrated secretion buffer. A mixture of the phosphodiesterase inhibitor 3-isobutyl-1-methylxanthine (IBMX) (Sigma, Poole, UK) and the adenylyl cyclase activator forskolin (Sigma, Poole, UK) at a final concentration of 10μM each were used as a positive control in secretion experiments. Cells were then incubated with 300 μl per well of test reagents for 2 hours at 37°C, 5%CO2/95% O2. Following incubation, supernatants were removed and centrifuged at 100 g for 3 minutes to remove cell debris. The resulting supernatant was then stored at -20°C before analysis. Cell lysis buffer was added to cells (250 μl/well) which were kept at -80ºC overnight. Plates were then scraped, washed with 250μl of secretion buffer and the lysates stored at -20°C pending analysis. Gut hormones were measured by radioimmunoassay (RIA) and secretion expressed as a fraction of the total peptide (secreted plus intracellular) measured in each well.

*ICV cannulation*

Rats were stereotaxically implanted with 22-gauge stainless steel cannulas in the lateral ventricle (LV) using coordinates determined from the Paxinos and Watson atlas ([5](#_ENREF_5)): 0.8 mm caudal and 1.5 mm lateral from the bregma and 3.2 mm below the dura. Correct positioning of cannula in the cerebroventricles was verified by a positive orexigenic response to neuropeptide Y (NPY) (2.5 nmol) administered in the early light phase. Animals consuming less than 2 grams of food in the first 2 hours following NPY administration were excluded from the study.

**Supplementary Results**

*The effect of OG administration of L-Arg on behaviour in rats*

Oral gavage administration of 16 mmolkg^-1^ L-Arg had no significant effect on measured behaviours compared to the water control group during 0-1 hour period following administration (Supplementary Figure 1).

*The effect of L-Arg on glucose homeostasis in GPRC6-a-KO mice*

Oral gavage of 4 mmolkg^-1^ L-Arg significantly reduced plasma glucose following a glucose tolerance test in both WT and GPRC6a-KO mice suggesting that this effect of L-Arg on insulin secretion does not require GPRC6A activity (Supplementary Figure 2). Two other GPRC6A knockout mice have been developed to investigate the physiological role of GPRC6A. The model reported by Pi *et al* was generated by targeted deletion of exon II which encodes for a minor part of the venus fly trap domain of the receptor which contains an orthosteric binding site for endogenous ligands ([6](#_ENREF_6)). Pi *et al* demonstrated that l-Arg-mediated insulin release in β-cells is mediated through GPRC6A activation of cAMP pathways ([7](#_ENREF_7)). However, another GPRC6A knockout mouse generated by disruption of exon VI, containing the entire 7 transmembrane and C-terminal region of the receptor ([8](#_ENREF_8)), has been reported to have normal glucose tolerance ([9](#_ENREF_9)). The GPRC6A knockout model used in our studies has the entire GPRC6a gene deleted. We found no differences in basal glucose levels or in the response to an IPGTT between WT and our GPRC6a knockout model ([10](#_ENREF_10)). In addition, L-Arg improved glucose tolerance in both WT and GPRC6a-KO animals, suggesting that this effect is not mediated via the GPRC6A (Supplementary Figure 4). Further work is required to understand the discrepancies reported between current GPRC6a knockout models.

*The role of GPRC6A in mediating L-Arg-induced gut hormone release in vitro*

Exposure to 100mM L-Arg significantly stimulated the release of GLP-1 from primary WT L-cells. GLP-1 levels were also elevated in GPRC6a-KO primary L-cells treated with L-Arg, though this effect did not reach statistical significance (Supplementary Figure 3A). Treatment of WT L-cells with 100mM L-Arg significantly stimulated PYY release from both WT L-cells and GPRC6a-KO L-cells (Supplementary Figure 3B).

*The effect of specific doses of GLP-1 and Y2 receptor antagonists on the effect of exogenous exendin-4 and PYY on food intake in mice*

IP administration of 1nmolkg^-1^ exendin 4 significantly reduced food intake in the first hour following administration. IP injection of 400nmolkg^-1^ of exendin 9-39 significantly attenuated the effect of exendin 4 on food intake (Supplementary Figure 4A). IP administration of 25nmolkg^-1^ PYY_3-36_ significantly reduced food intake in the first hour following administration. IP injection of 5.26μmolkg^-1^ of BIIE-0246 significantly attenuated the effect of exogenous PYY_3-36_ on food intake (Supplementary Figure 4B).

**Supplementary Tables**

| **Feeding study (Figure)** | **Species** | **Study phase** | **Feeding state** | **Route of administration** | **Dose of L-Arg (mmol/kg)*** |
| --- | --- | --- | --- | --- | --- |
| 1A | Rats | Light | Fasted | Oral gavage | 8, 16 |
| 1B | Rats | Dark | ad libitum | Oral gavage | 8. 16 |
| 1C | Mice | Light | Fasted | Oral gavage | 8, 16, 24 |
| 1D | Mice | Light | ad libitum | Oral gavage | 24 |
| 1E | Mice | Dark | ad libitum | Oral gavage | 24 |
| 1F | DIO Mice | Dark | ad libitum | Oral gavage | 16 |
| 2A, 2B | GPRC6a-KO Mice | Dark | ad libitum | Oral gavage | 16, 24 |
| 2C | SDA Rats | Light | Fasted | Oral gavage | 16 |
| 4A, 4B | Mice | Light | Fasted | Oral gavage | 24 |
| 4B | Mice | Dark | ad libitum | Oral gavage | 24 |
| 5A | Rats | Light | Fasted | Intraperitoneal | 4, 8 |
| 5B | Mice | Light | Fasted | Intraperitoneal | 4, 8, 12 |
| 5C | Rats | Light | Fasted | Intracerebroventricular | 4 µmol |

**Table S1.**

Table 1. Feeding studies conditions. Table details the experimental conditions including species, phase of the study, feeding state of the cohort, route of administration and the dose of L-Arg used for each of the feeding studies described in the manuscript. The Feeding study (Figure) column refers to the figure number showing the results for each study in the main manuscript. * All the doses presented in mmolkg^-1^ with exception of the ICV study where the dose is given in µmol.

**Supplementary Figures**

**Figure S1.**


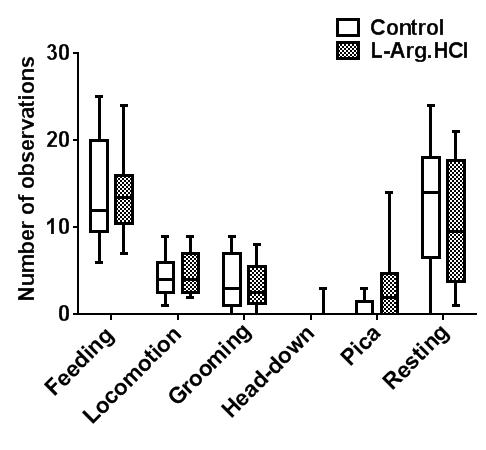


**Supplementary Figure S1**. **The effects of oral administration of L-Arg on behaviour in fasted rats during the light phase.** The effect of OG of water (control) or 16 mmolkg^-1^ L-Arg in overnight fasted male rats on feeding, locomotion, grooming, head down, pica and resting behaviours compared to control group. Data represented as median (interquartile range) for each observation. n=12-13.

**Figure S2.**


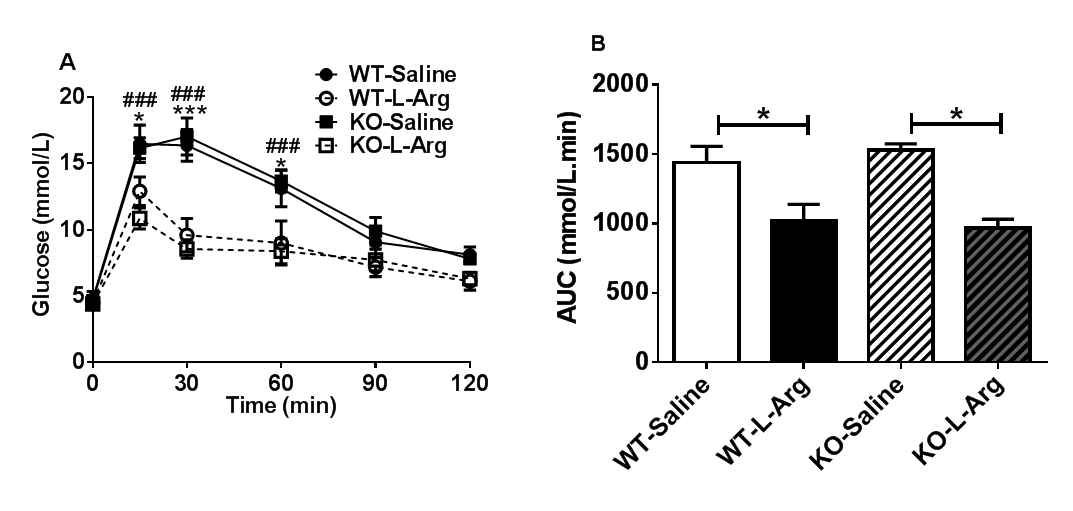
**Supplementary Figure S2**. The effect of L-Arg on glucose homeostasis in GPRC6a-KO mice. Glucose tolerance test (GTT) in female WT and GPRC6a-KO mice (A) and the area under the curve for each treatment (B). Mice were fasted overnight and received an intraperitoneal injection of 20% glucose solution (2g/kg body weight) followed by an immediate OG of 4 mmolkg^-1^ L-Arg. Data is presented as mean ± SEM. n=6 per group. **P*<0.05, ****P*<0.001 WT-L-Arg vs. WT-L-saline; ###P<0.001 vs. GPRC6a-KO-L-Arg vs. GPRC6a-WT.

**Figure S3.**


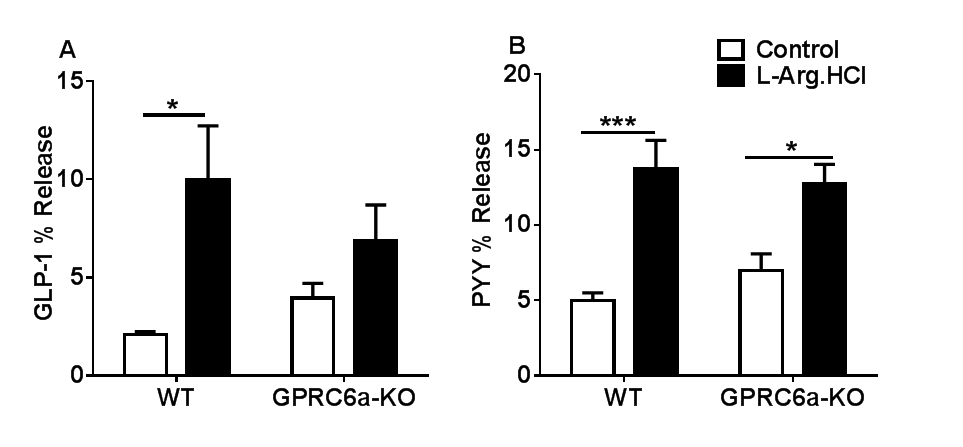


**Supplementary Figure S3**. **The effect of GPRC6A on GLP-1 and PYY release from primary murine colonic epithelium.** The effect of L-Arg on GLP-1 (A) and PYY (B) release from WT and GPRC6A-KO primary mice colonic L-cells incubated with 100mM L-Arg for 2h. The release is shown as percentage of total hormone contained for each well in the experiment. Data is presented as mean ± SEM. n=6 plates, from 6 mice. *p<0.05, ***p<0.001 vs. control.

**Figure S4.**


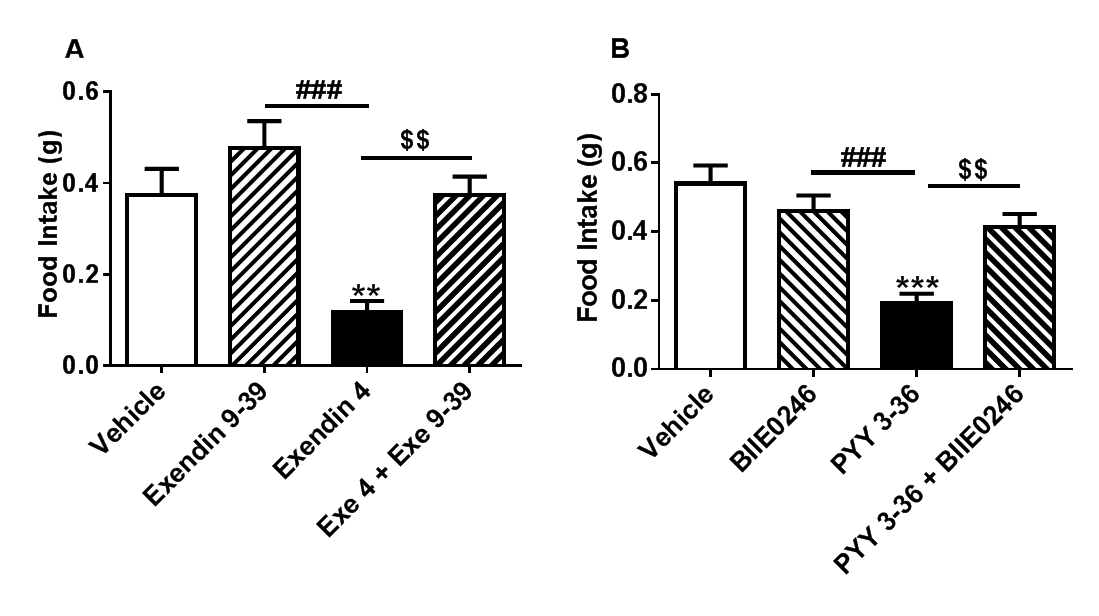


**Supplementary Figure S4**. **The effect of GLP-1 and Y2 receptors antagonism on the effects of exogenous exendin-4 and PYY on food intake in mice.** The effect of IP administration of 400nmol/kg exendin 9-39 on the anorectic effect of 1nmol/kg exogenous exendin-4 in fasted mice at 0-1h post administration (n=10) (A). The effect of IP administration of 5.26μmol/kg BIIE0246 on the anorectic effect of 25nmol/kg PYY(3-36) in fasted mice at 0-1h post administration (n=10) (B). Data is presented as mean ± SEM. (A): ***P*<0.01 vs saline control, ###*P*<0.001 vs. exendin 9-39, $$*P*<0.01 vs. exendin-4; (B): ****P*<0.001 vs vehicle control, ###*P*<0.001 vs. BIIE0246, $$*P*<0.01 vs. PYY(3-36).

**Supplementary references**

1. Ghourab S, Beale KE, Semjonous NM, Simpson KA, Martin NM, Ghatei MA, et al. Intracerebroventricular administration of vasoactive intestinal peptide inhibits food intake. Regulatory peptides. 2011;172(1-3):8-15.

2. Clemmensen C, Smajilovic S, Smith EP, Woods SC, Brauner-Osborne H, Seeley RJ, et al. Oral L-arginine stimulates GLP-1 secretion to improve glucose tolerance in male mice. Endocrinology. 2013;154(11):3978-83.

3. Williams DL, Baskin DG, Schwartz MW. Evidence that intestinal glucagon-like peptide-1 plays a physiological role in satiety. Endocrinology. 2009;150(4):1680-7.

4. Ghitza UE, Nair SG, Golden SA, Gray SM, Uejima JL, Bossert JM, et al. Peptide YY3-36 decreases reinstatement of high-fat food seeking during dieting in a rat relapse model. The Journal of neuroscience : the official journal of the Society for Neuroscience. 2007;27(43):11522-32.

5. Paxinos G., Watson C. The rat brain in stereotaxic coordinates. New YorK: Academic Press; 1998.

6. Pi M, Chen L, Huang MZ, Zhu W, Ringhofer B, Luo J, et al. GPRC6A null mice exhibit osteopenia, feminization and metabolic syndrome. PloS one. 2008;3(12):e3858.

7. Pi M, Wu Y, Lenchik NI, Gerling I, Quarles LD. GPRC6A mediates the effects of L-arginine on insulin secretion in mouse pancreatic islets. Endocrinology. 2012;153(10):4608-15.

8. Wellendorph P, Johansen LD, Jensen AA, Casanova E, Gassmann M, Deprez P, et al. No evidence for a bone phenotype in GPRC6A knockout mice under normal physiological conditions. Journal of molecular endocrinology. 2009;42(3):215-23.

9. Smajilovic S, Clemmensen C, Johansen LD, Wellendorph P, Holst JJ, Thams PG, et al. The L-alpha-amino acid receptor GPRC6A is expressed in the islets of Langerhans but is not involved in L-arginine-induced insulin release. Amino acids. 2013;44(2):383-90.

10. Kinsey-Jones JS, Alamshah A, McGavigan AK, Spreckley E, Banks K, Cereceda Monteoliva N, et al. GPRC6a is not required for the effects of a high-protein diet on body weight in mice. Obesity. 2015;23(6):1194-200.
